# Partial enrichment of phospholipids by enzymatic hydrolysis and membrane filtration of whey protein phospholipid concentrate

**DOI:** 10.3168/jdsc.2022-0322

**Published:** 2023-02-24

**Authors:** A.V. Swaminathan, M.S. Molitor, K.J. Burrington, D. Otter, J.A. Lucey

**Affiliations:** 1 Department of Food Science, University of Wisconsin, Madison 53706; 2 Center for Dairy Research, University of Wisconsin, Madison 53706

## Abstract

Whey protein phospholipid concentrate (WPPC) contains high amounts of phospholipids (PL; 4.5 ± 1%) but there is interest in further enriching the PL content for nutritional and functional applications. Chemical methods were unsuccessful in separating PL from proteins due to the presence of protein-fat aggregates. Instead, we explored hydrolysis of the proteins to peptides with the objective of removing peptides, thereby concentrating the PL fraction. We used microfiltration (MF) with a pore size of 0.1 µm to help reduce protein/peptide retention. Hydrolyzing proteins should facilitate passage of low molecular weight peptides through the MF membrane, while concentrating fat and PL in the MF retentate. Bench-top experiments were performed to select the proteolytic enzyme that resulted in the most extensive hydrolysis of proteins in WPPC from among 5 different commercial proteases. Sodium dodecyl sulfate-PAGE analysis was performed to measure the extent of protein hydrolysis over a period of 4 h. Alcalase enzyme was found to exhibit the highest proteolytic activity at conditions of pH 8 and temperature 55°C. The intensity of major protein bands (milkfat globule membrane proteins, caseins, β-lactoglobulin) in WPPC decreased in sodium dodecyl sulfate-PAGE profiles as hydrolysis progressed, along with the appearance of low molecular weight bands. Pilot-scale MF production, coupled with diafiltration (DF), of the hydrolyzed sample aided in the removal of peptides that caused an ~18% reduction in protein content with the final retentate having a total PL content of 9.3% dry basis (db) with protein and fat contents at approximately 43.8 ± 0.4% (db) and 48.9 ± 1.2% (db), respectively. The MF permeate had minimal fat content, indicating that there was no transmission of lipids or PL through the membrane during the MF/DF process. Confocal laser scanning microscopy and particle size analysis of enzyme hydrolyzed solution revealed that protein aggregates were still present after 1 h of hydrolysis. Complete removal of proteins and peptides was not achieved by this process, suggesting that a combination of enzymes would be needed for further hydrolysis of protein aggregates in WPPC solution to further enrich the PL content.

## Highlights

• Comparison of 5 protease enzymes was performed to determine the most proteolytic on WPPC.
• Alcalase was found to be the most proteolytic as analyzed through SDS-PAGE analysis.
• Microfiltration (MF) proved more successful in removing peptides and concentrating PL compared with ultrafiltration.
• Enriched PL concentrate with 9.3% PL (dry basis) was obtained by enzymatic hydrolysis and MF.

## Graphical Abstract

**Figure:**
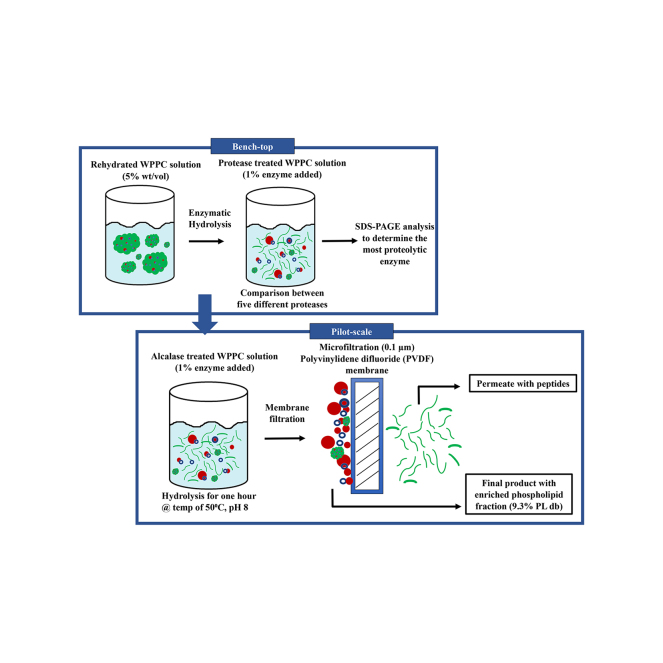
**Summary:** Bench-top and pilot-scale experiments were conducted to produce an enriched phospholipid (PL) fraction from whey protein phospholipid concentrate (WPPC). Five different protease enzymes were used individually for the enzymatic hydrolysis of proteins in a rehydrated WPPC solution (5% wt/vol). Alcalase exhibited the most proteolytic activity as determined using sodium dodecyl sulfate-polyacrylamide gel electrophoresis (SDS-PAGE) profiles and was therefore chosen for the pilot-scale experiment. Alcalase enzyme hydrolyzed most of the large protein aggregates (20-150 μm) present in the WPPC solution into smaller protein aggregates (0.3-20 μm) and small peptides helping the release of some of the fat and PL from the protein aggregates. Microfiltration (polyvinylidene fluoride membrane with 0.1-μm pore size) along with diafiltration was used to remove peptides from the hydrolyzed protein solution via the permeate and to concentrate the PL and fat to produce a partially enriched PL concentrate with 9.3% PL (dry basis) in the final product.

Phospholipids (**PL**) are of growing interest because of their nutritional and functional properties. Since the early 1900s, PL have been known to have beneficial effects concerning conditions like heart disease, inflammation, and cancer (Contarini and Povolo, 2013). There have been several research studies to separate and concentrate the PL from various dairy streams, such as milk, cheese whey, buttermilk, whey buttermilk, and butter serum.

Whey protein phospholipid concentrate (**WPPC**) is an underutilized dairy stream that was targeted for PL concentration; WPPC is obtained as a co-product when microfiltration (**MF**) is used to remove residual lipids from whey during manufacture of whey protein isolate (**WPI**). Removing residual lipids from whey not only helps with concentrating proteins but also has other advantages: (1) improves UF permeate flux during WPI processing, (2) prevents development of stale whey off-flavors derived from lipid oxidation, and (3) improves the functionality (emulsification and whipping properties) of WPI. Over the years improved membranes have been manufactured, and spiral-wound crossflow MF membranes are currently the most widely used membrane in the dairy industry for defatting purposes in the production of WPI (Kelly, 2019). The permeate from the MF of cheese whey has high levels of native whey proteins, and these proteins are further concentrated using UF and then spray dried to produce WPI powder, which is used for many highly functional food applications. The retentate from this MF process contains most of the residual lipids and PL, along with protein aggregates (mostly denatured). This product (liquid WPPC) can be spray dried to produce WPPC powder. Whey protein phospholipid concentrate has elevated concentrations of PL, about 5% of the product, and could potentially be a good starting material for further PL concentration.

Our previous study (Swaminathan et al., 2021) attempted to separate PL from WPPC using various chemical pretreatment methods (e.g., isoelectric precipitation of proteins, thermocalcic aggregation, and calcium-induced aggregation of whey proteins). All of these treatments were previously successful for defatting purposes when used on cheese whey and buttermilk (Rombaut et al., 2006; Rombaut and Dewettinck, 2007) but were not successful on WPPC solutions mainly because the protein and fat in WPPC were closely associated (aggregated) with one other. Confocal laser scanning microscopy (**CLSM**) images revealed the presence of very large protein aggregates containing entrapped fat, and this complex matrix of protein and entrapped fat makes PL extraction difficult by simple chemical treatments. Fractionation techniques, such as solvent extraction or enzymatic hydrolysis (**EH**), should be more effective in concentrating PL. Price et al. (2018) used a solvent (ethanol) extraction method to fractionate the PL in liquid WPPC. Their PL-enriched product had a total PL content of 45.8% dry basis (**db**). Solvent extraction methods are not common in the dairy industry and some solvents are not food grade. A different approach is EH of residual proteins in WPPC. Barry et al. (2017a) applied EH with subsequent UF (to remove peptides) to enrich the PL from reconstituted buttermilk powder (**BMP**). The results from this study showed that Alcalase enzyme (**AE**) was the most proteolytic among the 3 proteases used, generating peptides of <50 kDa (about 89% of the total peptides). Ultrafiltration in combination with this treatment proved successful in reducing the protein content from 31.4 ± 0.57% to 17.9 ± 0.19% in the retentate while increasing the lipid and PL content from 6.84 ± 0.17% to 43.4 ± 0.61% and 0.79 ± 0.00% to 6.16 ± 0.02%, respectively. A similar process was applied by Konrad et al. (2013) to whey buttermilk to concentrate PL. These authors performed EH of whey proteins using trypsin and pepsin and experimented with different molecular weight cut-offs, temperature, and pressures during UF processing. They found that a 2% degree of protein hydrolysis was enough to break down whey proteins into smaller peptides, and they were able to produce a concentrate with a purity of 14% PL. Enrichment of PL from WPPC has not been tried using EH and membrane filtration process, and we believe that a similar approach on WPPC can aid in concentrating the PL content.

Our objective was to evaluate the most proteolytic enzyme among 5 different proteases, which were then used for pilot-scale production of an enriched PL concentrate from WPPC. Both UF and MF membranes have been used in the past for the enrichment of PL from different products after various chemical pretreatments (Rombaut et al., 2006; Morin et al., 2007). Barry et al. (2017a) and Konrad et al. (2013) used UF in their process to retain most of the PL after EH. Our study evaluated both UF and MF membranes. Ultrafiltration retains all of the fat and PL but may limit the easy removal of peptides from the solution. Microfiltration has the ability to remove most of the peptides in solution, but there is a possibility of losing fat and PL via the permeate due to the larger pore size of this membrane. Therefore, a comparison between UF and MF membranes was performed for the most effective removal of peptides from the hydrolyzed solution to obtain the highest possible PL content in the final retentate product, without any fat or PL losses.

Spray-dried WPPC powder was obtained from Agropur Inc. The protease enzymes were sourced from Novozymes and DSM. Maxipro enzymes were obtained from DSM, and Alcalase, Flavorzyme, and Neutrase from Novozymes. The optimum conditions for these enzymes are reported to be within the pH range of 5.6 to 10.0 with an optimal temperature of 30°C to 60°C.

The WPPC powder was dissolved in Milli-Q water to prepare 5% (wt/vol) WPPC solutions for bench-scale experiments. These solutions were stirred for 2 h at room temperature and later refrigerated at 4°C for overnight rehydration. Enzymatic hydrolysis of proteins in WPPC solution was performed using 5 protease enzymes. One percent enzyme (% wt/wt, protein) was added to the WPPC solution after bringing the solution temperature and pH to optimum conditions of respective enzymes. Samples were incubated in a water bath for 4 h and samples were drawn at 8, 30, 60, 120, and 240 min to determine the extent of hydrolysis over time. Hydrolysis was stopped by inactivating the enzyme by heating the solution at 90**°**C for 10 min. The extent of hydrolysis achieved by each enzyme at different time points during incubation was measured using SDS-PAGE.

All the chemicals used were purchased from Sigma, Fisher Scientific, and Bio-Rad Laboratories. The pilot-scale experiment was performed by using 94 L of 5% WPPC solution prepared by dissolving appropriate amounts of WPPC powder in reverse osmosis (**RO**) water. This solution was rehydrated overnight under constant stirring and the temperature of the solution was kept below 4°C by recirculating chilled water through the jacket of the feed tank. Enzymatic hydrolysis of proteins in the feed solution was performed by bringing the temperature and pH of solution to optimum conditions selected for AE (pH 8.0 and temperature approximately 50°C to 60°C) and 1% AE (% wt/wt of protein) was added to feed solution. While continuous stirring was occurring, hydrolysis was carried out for 1 h before inactivating the enzyme by heating the solution at 90°C for 10 min.

Two crossflow elements, model ST-2B-3838 containing a polyethersulfone UF membrane and model V0.1–2B-3838 containing a polyvinylidene difluoride MF membrane with pore sizes of 10 kDa and 0.1 µm, respectively, were purchased from Synder Filtration for pilot-scale membrane filtration experiment. Runs comparing 2 membranes were carried out to determine the most efficient membrane for peptide removal without any fat losses and thereby chosen for PL enrichment process. The UF and MF elements were operated in series with the crossflow passing through UF element first. The retentate from the UF element passed through the MF element next, and then, as is standard for a 2-pump system, the retentate stream splits with the majority flowing back to the inlet of the recirculation pump. The minority of retentate was removed through a retentate flow control valve in route back to the feed tank. Membrane filtration was carried out at ⁓17°C with a boost pressure (difference between inlet and outlet pressure) maintained at 100 kPa throughout the run. About 200 L of RO diafiltration water was used to wash out solids, primarily peptides, that could pass through the MF membrane. The filtration runs of WPPC hydrolyzed solution were carried out in duplicate (n = 2).

Compositional analysis was performed on rehydrated WPPC solution. Total protein, fat, ash, moisture of WPPC and protein, and fat content of the MF and UF permeates were determined according to standard AOAC methods (AOAC International, 2000). Total PL analysis, CLSM, and particle size analysis were performed as previously described (Swaminathan et al., 2021).

Sodium dodecyl sulfate-PAGE analysis was used to compare the extent of hydrolysis of proteins achieved in rehydrated WPPC samples by different protease enzymes. Sodium dodecyl sulfate-PAGE profiles were used to measure the pattern of protein hydrolysis, disappearance of the major protein bands including caseins, β-LG, and α-LA, and the appearance of low molecular weight peptide bands smaller than the α-LA band, and to identify the type and form of proteins/peptides present in retentate and permeate after membrane filtration during the pilot plant run. Sodium dodecyl sulfate-PAGE analysis was performed as described in Swaminathan et al. (2021). The intensity of the protein bands on the SDS-PAGE gel obtained with various protease enzymes, at different incubation times, was quantified using an electrophoresis image analysis software called GelAnalyzer [GelAnalyzer 19.1 (www.gelanalyzer.com) by Istvan Lazar Jr., PhD and Istvan Lazar Sr., PhD, CSc].

Five different protease enzymes were used at bench-top scale to compare the extent of protein hydrolysis in WPPC solution achieved by each of these enzymes. Sodium dodecyl sulfate-PAGE was performed under both reducing and nonreducing conditions to better understand the origin of peptides from the proteins in WPPC.

Under reducing conditions, β-LG, α-LA, caseins, and milk fat globule membrane (**MFGM**) proteins were observed in the WPPC sample before hydrolysis with AE. Hydrolysis resulted in substantial breakdown of caseins and MFGM proteins, but a slower breakdown was observed of the β-LG and α-LA bands under reducing conditions for SDS-PAGE profiles. Almost all intact protein bands, except β-LG and α-LA, quickly disappeared at t (time) = 8 min for AE-treated solution. As the hydrolysis time increased, the intensity of the β-LG band decreased further and there were more small peptides produced as indicated by the appearance of low molecular weight bands (<10 kDa; results not shown). As seen in Table 1, AE was able to hydrolyze about 75% of the β-LG at t = 120 min, but much less of α-LA (25% at t = 60 min). Alcalase enzyme is a serine endopeptidase (i.e., it hydrolyzes serine specific amino esters). The proteins in WPPC, mainly caseins and β-LG, have various serine residues on the protein molecules. Caseins also have a higher number of phosphorylated serine residues. In the presence of high levels of calcium and phosphate, this results in the formation of insoluble calcium phosphate precipitates called nanoclusters (Lucey and Horne, 2018). These phosphorylated casein clusters in casein micelles tend to resist EH due to steric hindrance (Boutrou et al., 2010). But most intact caseins were partially hydrolyzed by t = 8 min in our experiments. Presumably, serum or soluble caseins (nonmicellar) occur in cheese whey material that were then concentrated in the production of WPPC. It may be easier to hydrolyze these soluble caseins compared with casein micelles where the caseins are crosslinked by these nanoclusters.

**Table 1:** Relative intensities (%) of different protein bands as compared with control sample (caseins, β-LG, and α-LA) under reducing conditions obtained in hydrolyzed whey protein phospholipid concentrate samples using various protease enzymes, incubated at 50°C, and obtained at different hydrolysis time points (8, 30, 60, 120, and 240 min)^1^

Incubation time (min)	Alcalase (pH 8.0)			Flavorzyme (pH 7.0)			Maxipro NPU (pH 7.0)			Neutrase (pH 7.0)			Maxipro FPC (pH 6.0)		
	Caseins^2^	β-LG	α-LA	Caseins	β-LG	α-LA	Caseins	β-LG	α-LA	Caseins	β-LG	α-LA	Caseins	β-LG	α-LA
8	0	67	>100^3^	55	>100	>100	48	66	>100	35	86	>100	67	80	>100
30	0	46	78	43	≥100	>100	43	59	>100	35	87	>100	59	77	>100
60	0	35	74	59	77	>100	33	46	>100	43	66	>100	56	64	>100
120	0	25	79	49	69	>100	41	49	>100	38	54	>100	54	56	>100
240	0	25	93	47	66	>100	36	50	>100	32	60	>100	46	26	>100

1 Alcalase, Flavorzyme, and Neutrase were obtained from Novozymes; Maxipro enzymes were from DSM.

2 No casein bands were observed in Alcalase enzyme–treated solution under reducing conditions for SDS-PAGE.

3 Values >100% indicate other peptides were present with a similar molecular weight after hydrolysis.

Flavorzyme seemed to have very little effect in hydrolyzing proteins in WPPC as compared with other enzymes. Most hydrolysis happened in first 8 min (i.e., a 50% reduction in casein bands as compared with control sample as seen in Table 1). The MFGM bands did not change much during incubation. Flavorzyme is an exopeptidase (i.e., it cleaves the terminal end of peptide bond in the protein molecule). The relatively poor hydrolyzing ability of Flavorzyme on proteins in WPPC could be due to its inability to reach the terminal active sites on the substrate. Proteins in WPPC were in a highly aggregated form. Most of the terminal sites of peptide bonds could have been buried within these high molecular aggregates, making them inaccessible to the active site of the enzyme.

Maxipro NPU and Neutrase enzymes exhibited similar behavior for hydrolysis of proteins in WPPC. Significant breakdown of MFGM proteins and caseins was observed under reducing conditions (Table 1). Maxipro NPU exhibited slower breakdown of β-LG but little breakdown of α-LA, whereas Neutrase had little or no breakdown of β-LG and α-LA as indicated in Table 1. Maxipro FPC hydrolyzed many proteins in WPPC. Under reducing conditions, increasing breakdown of MFGM proteins, β-LG, and α-LA, with an increase in incubation time, was observed. The initial MFGM proteins bands completely disappeared, and the β-LG band almost disappeared at t = 240 min (i.e., only 26% of β-LG remained intact as shown in Table 1). Some initial breakdown of caseins had occurred by t = 8 min but this did not increase further with time. Maxipro FPC was able to hydrolyze proteins in WPPC due to its endo-specific action.

Comparing the proteolytic patterns of all these enzymes, AE seemed to hydrolyze more proteins in WPPC and had less high molecular weight unresolved bands. Barry et al. (2017b) also determined AE to be their most proteolytic enzyme on BMP as it generated the greatest number of smaller molecular peptides. Alcalase enzyme with 1 h of hydrolysis time was therefore chosen for pilot plant separation in our study since there was no significant breakdown of proteins after that (Table 1).

Pilot-scale EH using AE was performed for 1 h at 55°C and pH 8, after which the enzyme was thermally inactivated (90°C for 10 min) and the WPPC hydrolyzed sample was cooled to 15°C before membrane filtration. The flux of feed material into the filtration element was maintained at 75 L/min, and the inlet and outlet pressures were 0.25 MPa and 0.028 MPa, respectively. Fluxes of UF and MF permeate were 4.2 and 5.3 L/min, respectively. During the membrane filtration run, the TS contents in the MF and UF permeate were continuously monitored using a handheld refractometer. After analyzing permeates for TS content, we found that the MF membrane was more effective for removing more peptides. The MF permeate and UF permeate collected during the initial stages of diafiltration had a protein content of approximately 1.37% and 0.86% total protein (as is), respectively, at the beginning of first filtration trial. The protein contents give an indication of peptide content (protein analysis is a measure of nitrogen content and can therefore also be used as an approximate method to measure peptide content). There were also no fat losses observed in MF permeate. This was confirmed from visual clarity of MF permeate. Turbidity in permeates is usually caused by the presence of fat but our MF permeate was very clear. This was also quantitatively confirmed, and fat content was <0.01% in the MF permeate. The MF membrane chosen for this experiment had a large enough pore size (0.1 µm) to let peptides to pass through the permeate while retaining the fat in retentate. The UF membrane had a smaller pore size of 10 kDa and was not efficient in removing peptides compared with the MF membrane. Based on these observations, the MF membrane was chosen for further pilot-scale experiments to remove the hydrolyzed peptides and concentrate the fat and PL. This was achieved by recirculating the UF permeate to the feed tank while removing the MF permeate. Diafiltration was carried out by connecting the RO water line to the feed tank. The MF process was continued until TS in MF permeate (as indicated by the refractometer) was <0.1%, indicating that minimal further peptides were being removed through MF permeate.

Compositional data of 5% WPPC solution and final retentate were obtained after the combined process of EH and MF/DF process. Proportion of protein in the sample decreased from 60.8 to 43.8% (db), thereby changing the total fat content from 27.5 to 48.9% (db). Total PL as a percentage of sample changed from 4.5% in the control sample to 9.3% (db) in the final retentate, a ⁓2-fold increase in the total PL content. On a db, there was an 18% decrease in protein content, and thereby a 22% increase in lipid content in MF retentate compared with the starting material. The protein:fat ratio changed from 2:1 in the feed material to about 1:1 in the final retentate. From these results, we can conclude that some proteins in WPPC were hydrolyzed to smaller peptides by AE and removed via the permeate during the MF/DF process. Barry et al. (2017b) used a similar method to concentrate PL from BMP, and the total PL content in their final retentate was about 11.05 ± 0.02% (as a % of retentate sample).

Confocal laser scanning microscopy images of rehydrated 5% WPPC solution (control), AE-treated solution, and enriched PL concentrate (MF retentate) are shown in Figure 1. Control solution had protein aggregates ranging in size between 5 and 30 µm as observed in Figure 1a. In Figure 1b, it can be seen that there were still protein aggregates present in the solution even after 1 h of hydrolysis. The average size of the aggregates was around 5 to 10 µm. This indicated that the EH was incomplete, or there was formation of aggregates during thermal inactivation of the enzyme as described by Barry et al. (2017a). The microstructure of the MF retentate looked very similar to the liquid WPPC sample from manufacturer 1 as reported by Swaminathan et al. (2021). Free fat fraction in MF retentate looks much bigger and coalesced as compared with the fat in liquid WPPC sample (Figure 2c).

**Figure 1:**
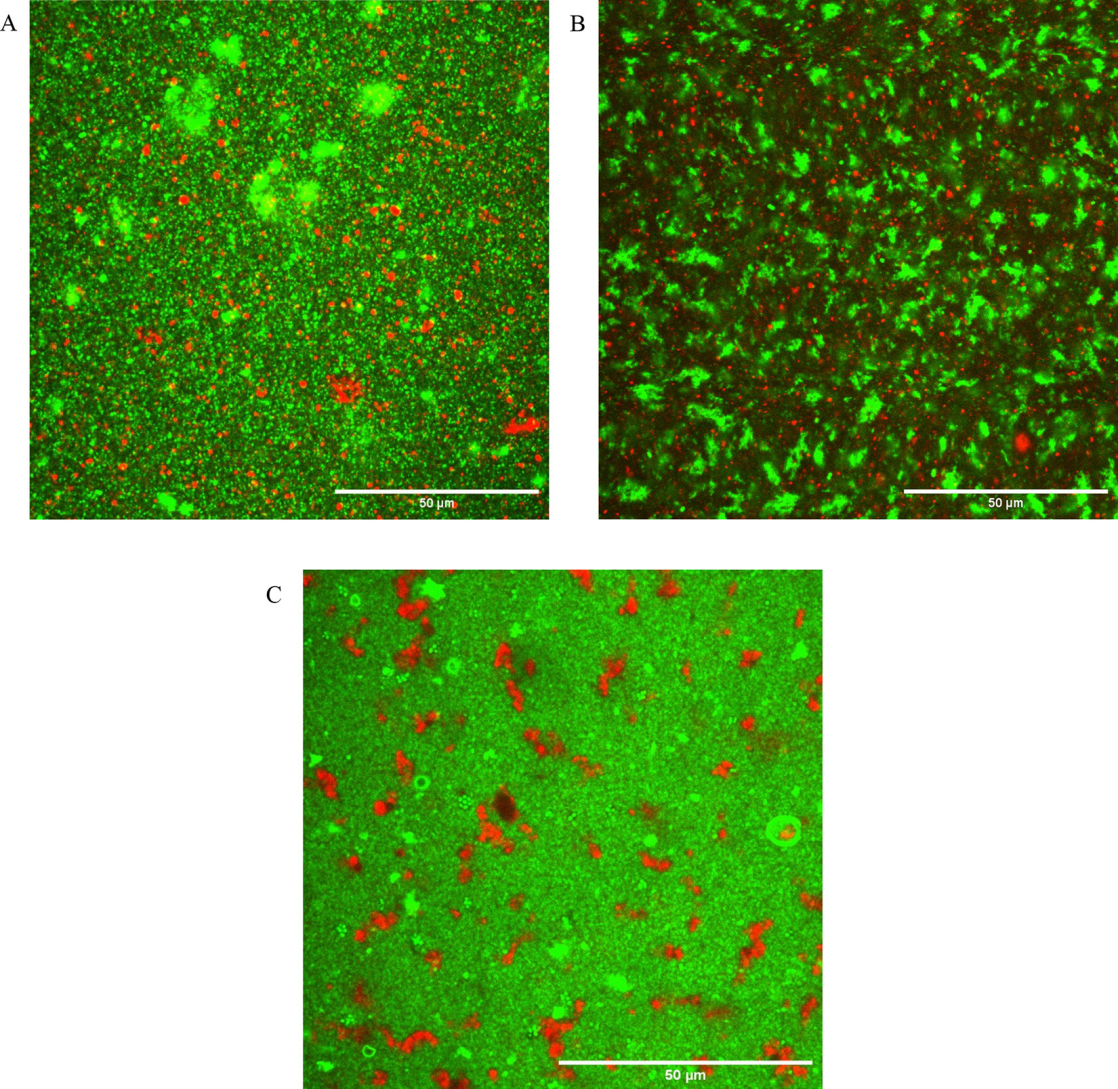
Confocal laser scanning microscopy images of (A) rehydrated 5% whey protein phospholipid concentrate (WPPC) solution (control), (B) Alcalase enzyme (Novozymes)–treated solution, and (C) enriched phospholipid concentrate. Scale bar = 50 µm. Proteins are stained green with Fast Green FCF (Sigma-Aldrich), and lipids are stained red with Nile Red (Sigma-Aldrich).

**Figure 2:**
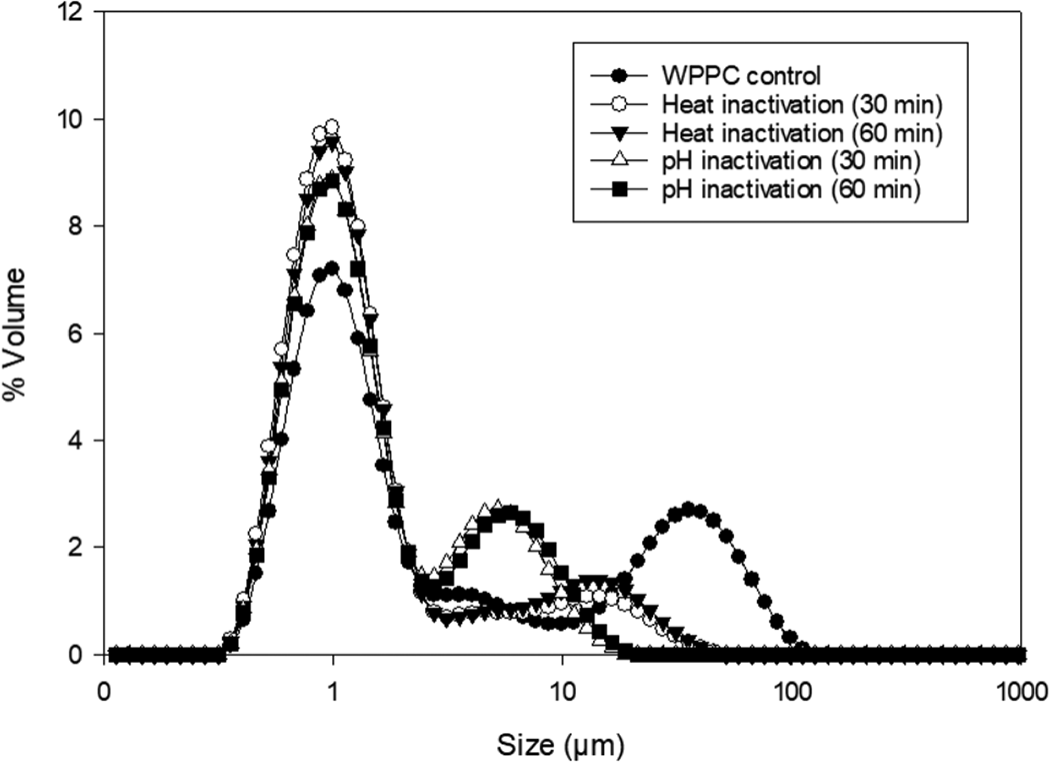
Particle size distribution of rehydrated 5% whey protein phospholipid concentrate (WPPC) solution, Alcalase enzyme (Novozymes)–treated solution after 30 min and 1 h of hydrolysis that were subjected to either thermal inactivation or pH change to inhibit activity of enzyme.

Particle size analysis was performed to investigate if there was aggregate formation due to high temperatures used for thermal inactivation of enzymes. For this experiment, the WPPC solution hydrolyzed with AE (30 min and 1 h) was subjected to either thermal inactivation of enzyme (90°C for 10 min) or the solution pH was adjusted to 5 using 0.5 *N* HCl to inhibit the activity of the enzyme. Particle size analysis was performed on both these solutions to observe the size of aggregates present in solution. It was seen that thermally inactivated enzyme solutions had larger aggregates (5–50 µm) compared with pH-adjusted solution (5–15 µm) but the % volume contributions of the aggregates in thermally treated solutions were lower as compared with aggregates in pH adjusted solution. Although there were differences in aggregate formations between heat-treated and pH-adjusted solution, both solutions had aggregates that were significantly higher than the MF membrane pore size (0.1 µm) used for pilot-scale membrane filtration. It can be seen in Figure 2 that there were no differences in particle size between the solutions that were allowed to hydrolyze for 30 min and 1 h. This suggests that EH happened very fast in the initial 30 min and slowed down markedly after that. This sudden decrease in hydrolysis might be due to the pH drop in solution as hydrolysis progressed (pH of the solution as measured at 0, 15, 30, 45, and 60 min after the enzyme added was 7.99, 6.56, 6.48, 6.46, and 6.41, respectively) or it might be because the maximum % degree of hydrolysis (**max % DH**) was already reached by the enzyme (max % DH is 15–25% for AE as reported by the manufacturer). The WPPC solution was not maintained under optimum pH conditions for the enzyme because the activity of AE is still about 80% even at pH 6.5 (Zeng et al., 2020). This suggested that the incomplete hydrolysis of proteins was because the enzyme had reached max % DH.

The PL enrichment using EH and membrane filtration worked slightly better in BMP (Barry et al., 2017a) because it is a relatively easier system to work with. The BMP has all the proteins in milk, mostly in their original “native” form compared with WPPC. About 80% of the proteins in BMP are caseins, which are heat stable. Buttermilk powder also has about 50% lactose (db) that limits denaturation and aggregation during the concentration, evaporation, and spray drying process. The WPPC on the other hand has various proteins that form a very complicated matrix. The separated whey from cheesemaking has to undergo roughly a 100-fold concentration due to extensive MF and DF during which most of the soluble native serum proteins are removed via the permeate and used as feed for whey protein concentrate/isolate production. The MF retentate, which is the liquid WPPC, is a concentrated mass of mostly denatured, aggregated proteins. Liquid WPPC has about 60% protein and 30% fat (db) with little or no lactose. When this product further undergoes potential evaporation, heat treatment, or both before it is spray dried, the proteins aggregate because the proteins are prone to significant aggregation when concentrated. The resulting product when rehydrated in solution had huge protein aggregates (20–150 µm) with entrapped fat as observed by Swaminathan et al. (2021) in their CLSM images of control rehydrated 5% WPPC solution. The AE enzyme does a good job of hydrolyzing proteins, but it probably had limited access to active sites in our WPPC protein matrix.

The goal of our research was to develop a separation process to produce enriched dairy PL from WPPC. This goal was partially achieved by the combination of EH and MF/DF process. Among the 5 different protease enzymes, AE was determined to be the most proteolytic enzyme for our sample based on the SDS-PAGE results obtained with the bench-top experiments. A combination of EH and a MF/DF process was able to produce a final product with a phospholipid content of 9.3% (% of final retentate in db) with protein and fat content at about 44% and 49% (db), respectively. A more concentrated PL rich fraction could be produced by this separation process if a combination of enzymes was used to hydrolyze more of the protein fraction in WPPC. Further studies are required to evaluate the capabilities of using various enzymes in combination to efficiently hydrolyze the proteins to a much smaller size than the pore size of the membrane used for filtration (<0.1 µm).
